# P2X7 receptor antagonist improves gastrointestinal disorders in spontaneously hypertensive rats

**DOI:** 10.1590/1414-431X2023e12569

**Published:** 2023-02-27

**Authors:** K.B.V. de Oliveira, J.S. Severo, A.C.A. da Silva, B.L.B. dos Santos, P.H.M. Mendes, J.P.J. Sabino, A.L.M.M. Filho, P. Correia-de-Sá, A.A. dos Santos, M.T.B. da Silva

**Affiliations:** 1 Programa de Pós-Graduação em Farmacologia, Universidade Federal do Piauí, Teresina, PI, Brasil; 2 Programa de Pós-Graduação em Alimentação e Nutrição, Universidade Federal do Piauí, Teresina, PI, Brasil; 3 Departamento de Educação Física, Laboratório de Exercício e Trato Gastrointestinal, Universidade Federal do Piauí, Teresina, PI, Brasil; 4 Departamento de Biofísica e Fisiologia, Universidade Federal do Piauí, Teresina, PI, Brasil; 5 Programa de Pós-Graduação em Ciências Farmacêuticas, Universidade Federal do Piauí, Teresina, PI, Brasil; 6 Departamento de Fisiologia e Farmacologia, Faculdade de Medicina, Universidade Federal do Ceará, Fortaleza, CE, Brasil; 7 Centro de Ciências da Saúde, Universidade Estadual do Piauí, Teresina, PI, Brasil; 8 Departamento de Imuno-Fisiologia e Farmacologia, Laboratório de Farmacologia e Neurobiologia, Centro de Descoberta de Fármacos e Medicamentos Inovadores, Instituto de Ciências Biomédicas Abel Salazar, Universidade do Porto, Porto, Portugal; 9 Departamento de Imuno-Fisiologia e Farmacologia, Laboratório de Fisiologia, Centro de Descoberta de Fármacos e Medicamentos Inovadores, Instituto de Ciências Biomédicas Abel Salazar, Universidade do Porto, Porto, Portugal

**Keywords:** Angiotensin II, Hypertension, SHR, Oxidative stress, Gastrointestinal tract, Gastric emptying

## Abstract

The purinergic system participates in the control of blood pressure. Hypertension promotes the occurrence of gastrointestinal disorders such as intestinal inflammation and gastric emptying delay. This study aimed i) to investigate the participation of the P2X7 receptor blocker Brilliant Blue G (BBG) on gastric emptying of solids and changes in oxidative stress in the gastric fundus, duodenum, and colon of spontaneously hypertensive rats (SHR) and ii) to study the putative relationship of this effect with the renin-angiotensin system. Rats were divided into five groups: Control, SHR, SHR+BBG, SHR+BBG+ATP, and SHR+BBG+ANG II. In the gastrointestinal tract, we assessed gastric emptying (GE) and oxidative stress markers (NOx, MPO, GSH, SOD). We observed a decrease in the GE rate (P<0.05) in SHR *vs* control rats (21.8±2.0% *vs* 42.8±3.5%). The decrease in GE was returned (P<0.05) to control levels by BBG in SHR rats (21.8±2.0% *vs* 41.6±3.2%). Co-administration of ATP or ANG II together with BBG bypassed the effect of the P2X7 antagonist on GE in SHR (P<0.05) (21.9±5.0% *vs* 25.6±3.0% *vs* 41.6±3.2%). The MPO activity increased (P<0.05) in the gastric fundus of SHR compared to control rats (6.12±2.26 *vs* 0.077±0.02 UMPO/mg tissue); this effect was prevented (P<0.05) by BBG (0.55±0.15 *vs* 6.12±2.26 UMPO/mg tissue). Data demonstrated that blockage of P2X7 receptors with BBG can improve the GE delay and oxidative stress biomarkers in SHR animals. This preventive effect of BBG on GE delay was abrogated by ANG II and ATP, thus prompting crosstalk between renin-angiotensin and the purinergic signaling systems underlying this phenomenon.

## Introduction

According to the 2020 International Society of Hypertension Global Hypertension Practice Guidelines (1), systemic arterial hypertension (SAH) is a multifactorial disease whose diagnosis is based on elevated blood pressure, with systolic blood pressure (SBP) ≥140 mmHg and diastolic blood pressure (DBP) ≥90.

The effort to understand the mechanisms related to SAH has led to the development of numerous experimental models to simulate hypertensive responses. One of the models widely used today is the spontaneously hypertensive rat (SHR) model, which was initially described in 1963 (2). These animals are the most commonly used by researchers around the world. Their main characteristics are a genetic predisposition to hypertension and the ability to mimic essential arterial hypertension in humans (3). SHR animals show high blood pressure levels from 5 weeks of age, where systolic blood pressure can reach around 180-200 mmHg (2).

SHR animals exhibit overactivation of the sympathetic nervous system (SNS) resulting in alterations of the renal function, excess sodium reabsorption and renin secretion, and deficient natriuresis. Sympathetic overshooting may be associated with organic malfunctioning, most commonly including a baroreflex dysfunction (3).

Changes in purinergic receptor signaling responses are also involved in the pathophysiology of SAH (4). Overactivation of ATP-sensitive P2X receptors also participates in the long-term control of blood pressure, which involves mechanisms related to pressure natriuresis, autoregulation of glomerular filtration rate and blood flow, and regulation of sodium elimination (5). On the other hand, hypertensive-related changes in the sympathetic tone, purinergic signaling, and renin-angiotensin-aldosterone system (RAAS) may have significant repercussions in other organ systems, like the gastrointestinal (GI) tract. These changes included alternations in GI permeability and motility, inflammation, and oxidative stress (6).

The purinergic system can influence the motility, secretion, absorption, and permeability of the gastrointestinal tract. Purines released from intrinsic enteric nerves, sympathetic nerves, or sensory nerves during axon reflexes may act directly on epithelial cell receptors or on smooth muscle purinoceptors to mediate relaxation or contraction. Purines also act as autocrine mediators on prejunctional nerve terminals to modify the release of transmitters to control both motor and sensory nerve local reflexes. In this context, purines modulate synaptic transmission in both myenteric and submucosal ganglia to control GI motility and mucosal secretion and absorption (7).

The purinergic P2X7 receptor is an adenosine triphosphate (ATP)-gated ion channel found in many cell types, including immunocytes (e.g., lymphocytes, monocytes, and macrophages). It belongs to the membrane-bound inflammasome complex, which upon activation by ATP initiates post-translational modifications of interleukin 1 (IL-1) and IL-18, thus favoring their secretion and activity. Blockage of the P2X7 receptor has many pathophysiological repercussions, namely in the cardiovascular and gastrointestinal systems. Mounting evidence indicates that P2X7 receptor antagonists, like Brilliant Blue G (BBG), may be therapeutically beneficial in neurological and inflammatory diseases (8).

BBG is a derivative of a synthetic food-coloring compound that is considered toxic for humans perhaps because of its P2X7 antagonistic properties. Selective P2X7 receptor antagonists, like the CE-224,535, have been used in human clinical trials for the treatment of rheumatoid arthritis to reduce leukocyte secretion of IL-1ß and IL-18 (9). AZD9056, another P2X7 receptor antagonist, has been tested to improve symptoms in patients with moderate-to-severe Crohn's disease (10).

Oxidative stress causes an imbalance between the generation of reactive oxygen species (ROS) and reactive nitrogen species (RNS), causing their neutralization by the body’s defense mechanisms (11). In a previous study, Silva et al. (12) showed that hypertensive animals have high levels of oxidative stress markers and reduced antioxidant activity in the GI tract, thus having a tremendous impact on motility and neuronal function in this system.

In this context, this study was designed to fill a gap in our knowledge regarding the pathophysiology of gastric alterations in SAH and the putative beneficial impact of the purinergic system blockage and its relationship with the renin-angiotensin system on gastric emptying delay and oxidative stress alterations in SHR.

## Material and Methods

### Animals and ethical approval

Male Wistar rats were used as control animals and SHR rats (n=8-9/group; weight: 180-220 g) formed the treatment groups. All experiments were conducted according to “3R” principles. The SHR rats were donated by the Central Animal Facility of the State University of Piauí (UESPI, Brazil). The Wistar rats came from the Central Animal Facility of the Federal University of Piauí (UFPI, Brazil). The animals were kept in large standard cages (30×50×25 cm), duly identified, under monitored temperature (22±2°C), with water and food provided *ad libitum*, and in a 12-h light/dark cycle (light phase from 06:00 to 18:00 h). The animals were housed in the sectorial vivarium of the Medicinal Plants Research Center (NPPM), Block SG-15, UFPI.

**Figure 1:**
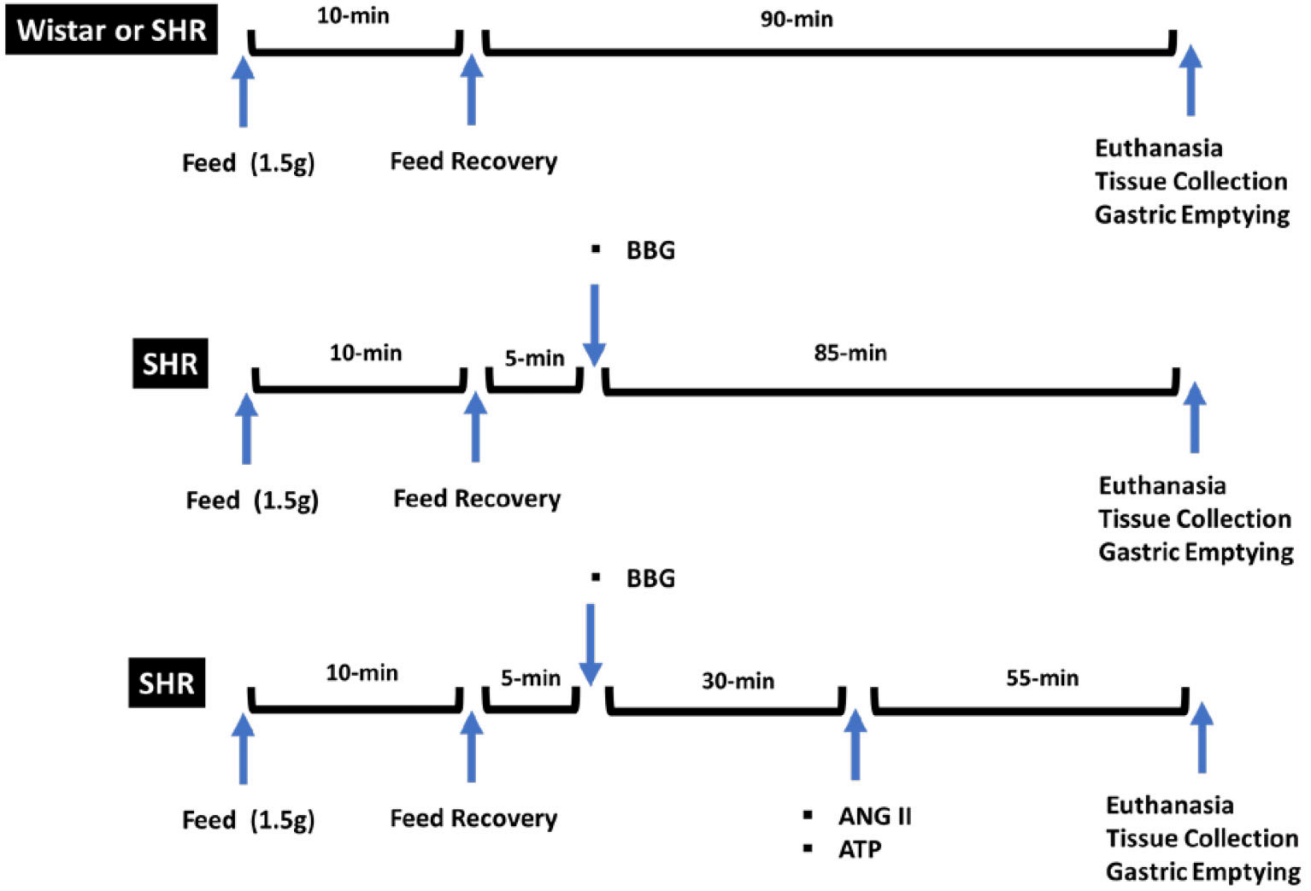
Experimental design. SHR: spontaneously hypertensive rat; BBG: Brilliant Blue G; ANG II: angiotensin II; ATP: adenosine triphosphate.

All procedures were performed under the recommendations of the Guide for the Care and Use of Laboratory Animals (US National Institutes of Health, 1996) after approval by the Ethics Committee on Animal Use (CEUA) of the Federal University of Piauí (Protocol 627/20). The rats were divided into five experimental groups: a normotensive control group, an SHR group, and three SHR treatment groups (SHR+BBG, SHR+BBG+ATP, SHR+BBG+angiotensin II). Figure 1 shows the experimental design of this study.

### Assessment of gastric emptying of solids

Gastric emptying (GE) of solids was analyzed according to the method described by Telles et al. (13). One week before the experimental protocols and the pharmacological intervention, Wistar or SHR rats were placed in individual cages with free access to water and feed in Petri dishes, for acclimation to the feeding site. Next, the animals fasted for 24 h. On an experimental day, the rats were allocated to individual boxes and received feed (1.5 g) in Petri dishes. After 10 min, the feed that was not consumed was removed from the cage and weighed, thus estimating the amount ingested by each rat by subtraction. In SHR groups, the feed was removed, and, after 5-min, the groups were treated with BBG (50 mg/kg, *sc*), a P2X7 antagonist (14). Thirty minutes after BBG, 2 separate groups received ANG II (100 μg/kg, *ip*) (15) or ATP (2 mg/kg, *ip*) (16). Ninety minutes after the treatments, the rats were sacrificed by an overdose of sodium thiopental (100 mg/kg, *ip*). Then, a median laparotomy was performed, so that the pylorus and cardia were clamped, thus allowing the removal of the stomach without altering the gastric content. After the stomach was removed, it was opened at the greater curvature and its contents were recovered. The recovered material was dried in an oven at a temperature of 100°C for 4 h, after which the content was weighed to calculate the gastric emptying according to the formula: Gastric emptying (%) = (1 - [dry weight of feed recovered from stomach / weight of feed intake]) × 100.

### Myeloperoxidase (MPO) analysis

Briefly, the tissues of the gastric fundus, duodenum, and colon were homogenized in potassium buffer with 0.5% hexyltrimethylammonium (HTAB) (1 mL/100 mg of tissue). Then, the homogenate was centrifuged at 2490 *g* for 20 min at 4°C. MPO activity in the resuspended pellet was evaluated by measuring the change in absorbance at 450 nm using o-dianisidine dihydrochloride and 1% hydrogen peroxide. The results are reported as units of MPO per mg of tissue (UMPO/mg of tissue) (12).

### Reduced glutathione (GSH) analysis

The concentration of GSH in the gastric fundus tissue, duodenum, and colon samples was analyzed according to the method described by Reilly et al., (17). To determine the levels of the non-protein sulfhydryl (NPSH) groups, samples between 50 to 100 mg of the animals’ gastric fundus were homogenized at a concentration of 1 mL of 0.02 M ED2 for each 100 mg of tissue. Aliquots of 400 μL of the homogenate were mixed in 320 μL of distilled water and 80 μL of 50% trichloroacetic acid (TCA) for protein precipitation to occur. Tubes containing the material were centrifuged for 15 min at 1107 *g* and 4°C. Then, 400 μL of the supernatant was added to 800 μL of 0.4 M Tris buffer (pH 8.9) and 20 μL of dithiol-nitrobenzoic acid (DTNB, or Ellman's reagent). Next, the mixture was stirred for 3 min and its absorbance was read by a spectrophotometer at 412 nm. The concentrations of the NPSH groups are reported in mg NPSH/mg tissue.

### Nitrite/nitrate (NOx) evaluation

The production of nitric oxide in the tissue gastric fundus, duodenum, and colon of the animals was indirectly evaluated by quantifying the levels of nitrate (NO_3_^-^) and nitrite (NO_2_^-^) (collectively referred to as NOx), using the Griess method. The samples were macerated in a potassium chloride solution (KCl, 0.15 M) and the homogenate was centrifuged at 12,000 *g* for 20 min and -4°C. Then, the supernatant (100 μL) was mixed with Griess reagent (100 μL) (phosphoric acid, sulfanilamide, and N-(1-naphthyl) ethylenediamine dihydrochloride). After 10-min, the absorbance of the samples was measured at 540 nm. The results are reported as micromoles of NOx (12).

### Superoxide dismutase (SOD) levels

Using gastric fundus, duodenum, and colon samples, a 10% homogenate was prepared and centrifuged at 1107 *g* for 15 min at 4°C. Subsequently, each sample was added to a solution of phosphate, L-methionine (20 mM), Triton X-100 (1% v/v), hydroxylamine chloride (10 mM), and EDTA (50 μM). The tubes were placed in a water bath at 37°C for 5 min. Riboflavin (50 μM) was added, and all measurements were corrected in a white light box for 10 min. The solution was then transferred to an ELISA plate, followed by the addition of the Griess reagent, performed in an ELISA reader at 550 nm. The value of the SOD unit (USOD/µg tissue) was calculated (12).

### Statistical analysis

Data are reported as means±SE. Subsequently, they were exported to the GraphPad Prism program (version 8; USA) for statistical analysis. The Kolmogorov-Smirnov test was applied to verify the normality of the data. Then, one-way ANOVA followed by the Tukey test was used to compare three or more groups. In other analyses, we used Pearson’s linear correlation coefficient for the association between GE and oxidative stress parameters. The difference was considered significant when P<0.05 (confidence interval of 95%).

## Results

**Figure 2:**
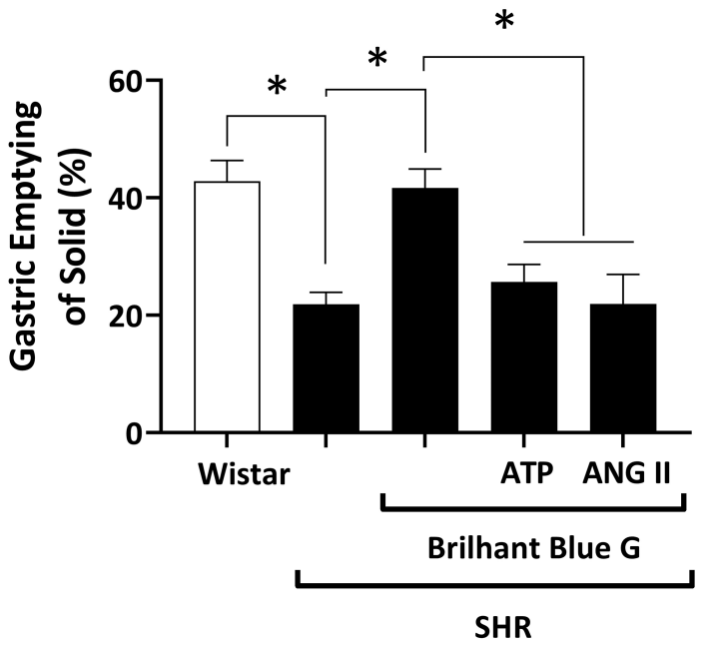
Gastric emptying of a solid test meal of Wistar *vs* spontaneously hypertensive rats (SHR), SHR treated with BBG, SHR treated with BBG plus ATP, and SHR treated with BBG plus ANG II. Data are reported as means±SE. *P<0.05 (one-way ANOVA, followed by the Tukey test). BBG: Brilliant Blue G; ANG II: angiotensin II; ATP: adenosine triphosphate.

Figure 2 shows the solid GE rate of the rats. There was a significant decrease (P<0.05) in solid GE in SHR compared to control animals (21.88±2.05% *vs* 42.81±3.57%). Subcutaneous administration of BBG reverted (P<0.05) the solid GE delay observed in SHR (21.88±2.05%) to a situation similar to that observed in control animals (41.66±3.26%). The beneficial effect of BBG on solid GE delay in SHR (41.66±3.26%) was abrogated by co-application of ATP (25.66±3.01%; P<0.05). A similar situation was observed when ANG II was used instead of ATP; the solid GE delay observed in the BBG+ANG II group of SHR (21.91±5.07%) was similar to that obtained in SHR without any treatment (21.88±2.05%).

**Figure 3:**
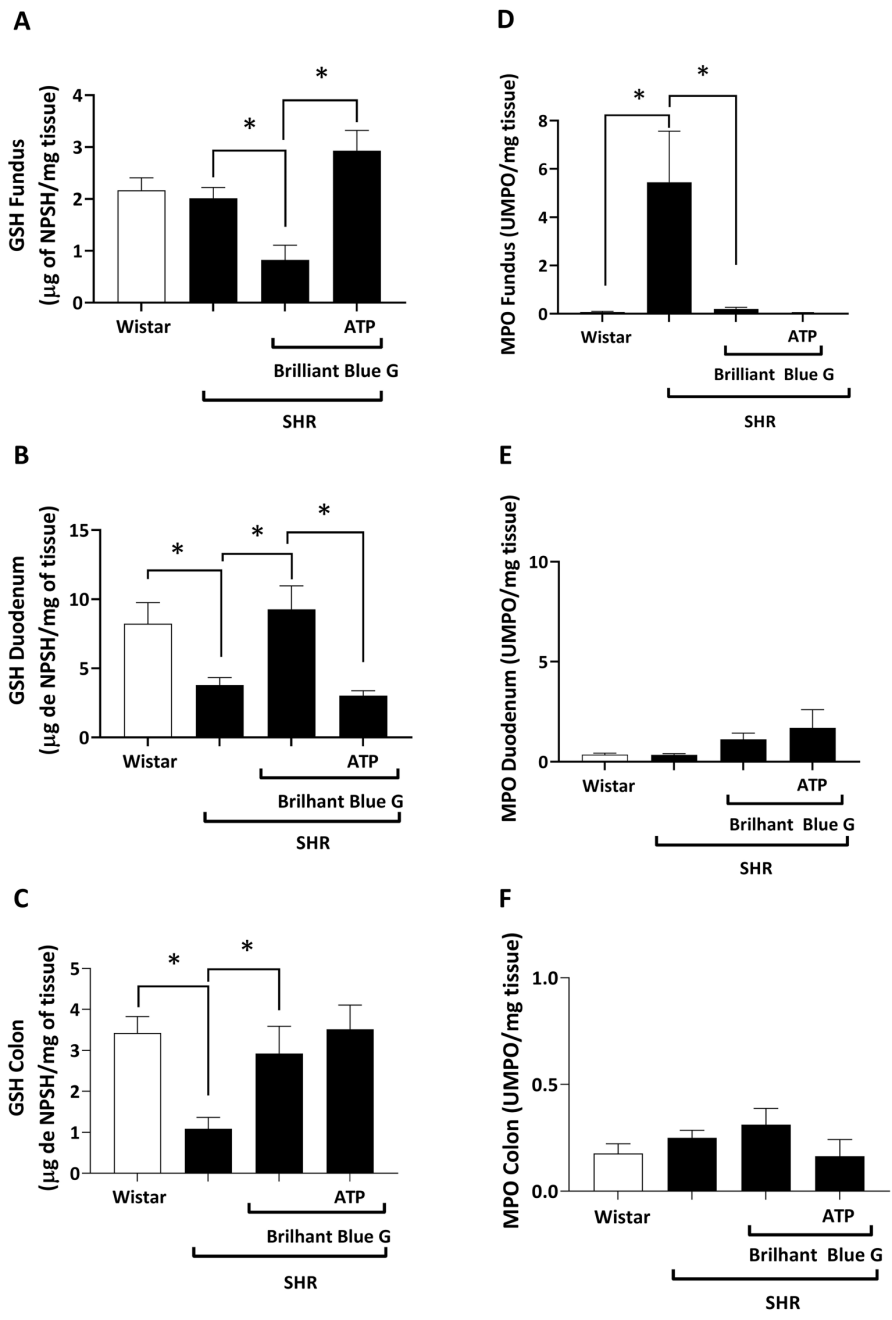
Glutathione (GSH) concentrations in the gastric fundus (**A**), duodenum (**B**), and colon (**C**) of Wistar rats *vs* spontaneously hypertensive rats (SHR), SHR treated with BBG, and SHR treated with BBG plus ATP. Myeloperoxidase (MPO) activity in the gastric fundus (**D**), duodenum (**E**), and colon (**F**) of Wistar rats *vs* SHR, SHR treated with BBG, and SHR treated with BBG plus ATP. Data are reported as means±SE. *P<0.05 (one-way ANOVA, followed by the Tukey test). BBG: Brilliant Blue G; ATP: adenosine triphosphate; NPSH: non-protein sulfhydryl; UMPO: units of MPO.

**Figure 4:**
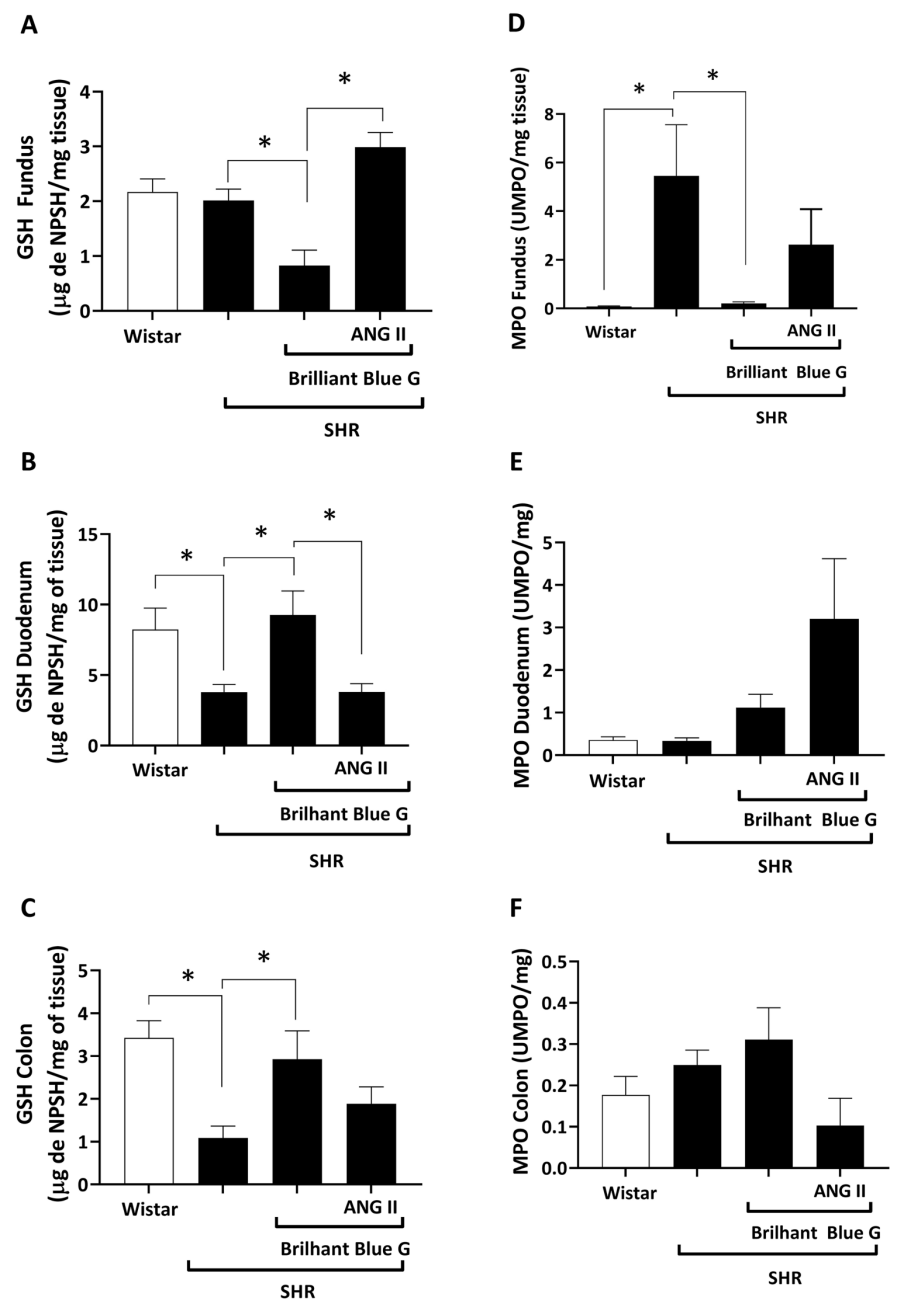
Reduced glutathione (GSH) concentrations in the gastric fundus (**A**), duodenum (**B**), and colon (**C**) of Wistar rats *vs* spontaneously hypertensive rats (SHR), SHR treated with BBG, and SHR treated with BBG plus ANG II. Myeloperoxidase (MPO) activity in the gastric fundus (**D**), duodenum (**E**), and colon (**F**) of Wistar rats *vs* SHR, SHR treated with BBG, and SHR treated with BBG plus ANG II. Data are reported as means±SE. *P<0.05 (one-way ANOVA, followed by the Tukey test). BBG: Brilliant Blue G; ANG II: angiotensin II; NPSH: non-protein sulfhydryl; UMPO: units of MPO.

Figure 3A shows that the concentration of GSH in the gastric fundus of the control group (2.167±0.2405 NPSH/mg tissue) was not significantly different (P>0.05) from that obtained in the SHR group (2.01±0.20 NPSH/mg tissue). Blockage of the P2X7 receptor with BBG reduced (0.82±0.28 NPSH/mg tissue; P<0.05) the pool of GSH detected in the gastric fundus of SHR animals (2.01±0.20 NPSH/mg tissue), but the level of GSH returned to higher than control levels when ATP was administered together with BBG (Figure 3A). Likewise, co-administration of ANG II+BBG increased (P<0.05) the concentration of GSH in the gastric fundus of SHR animals (2.99±0.26 NPSH/mg tissue) (Figure 4A).

SHR rats had significantly (P<0.05) lower levels of GSH in the duodenum (3.80±0.53 NPSH/mg tissue) and colon (1.08±0.28 NPSH/mg tissue) than control animals (8.23±1.53 and 3.42±0.40 NPSH/mg tissue, respectively) (Figure 3B and C). Treatment with BBG increased (P<0.05) the concentration of GSH in the duodenum (9.268±1.696 NPSH/mg tissue) and colon (2.928±0.661 NPSH/mg tissue) of SHR to levels comparable to those observed in control animals (Figure 3B and C). Co-administration of ATP+BBG reverted the preventive effect of BBG alone in the duodenum (3.02±0.35 vs. 9.27±1.69 NPSH/mg tissue) (Figure 3B), but not in the colon (Figure 3C), of SHR animals. ANG II had a similar effect as ATP; that is, the BBG-induced increase in the GSH concentration was reversed by ANG II in the duodenum (3.800±0.588 NPSH/mg tissue; Figure 4B), but not in the colon (1.88±0.39 NPSH/mg tissue; Figure 4C), of SHR animals.

The gastric fundus of SHR animals (Figure 3D), but not the duodenum (Figure 3E) and colon (Figure 3F), exhibited higher MPO activity than that observed in control rats (5.45±2.10 *vs* 0.07±0.02 UMPO/mg tissue; P>0.05). Blockage of the P2X7 receptor with BBG reversed the MPO activity in the gastric fundus of SHR to control levels (2.61±1.46 *vs* 0.20±0.07 UMPO/mg tissue), but we did not detect any significant (P>0.05) differences when BBG was co-administered with ATP (Figure 3D) or ANG II (Figure 4D). Surprisingly, ANG II (Figure 4E), but not ATP (Figure 3E), increased the MPO activity in the duodenum of SHR treated with the P2X7 receptor blocker, BBG.

**Figure 5:**
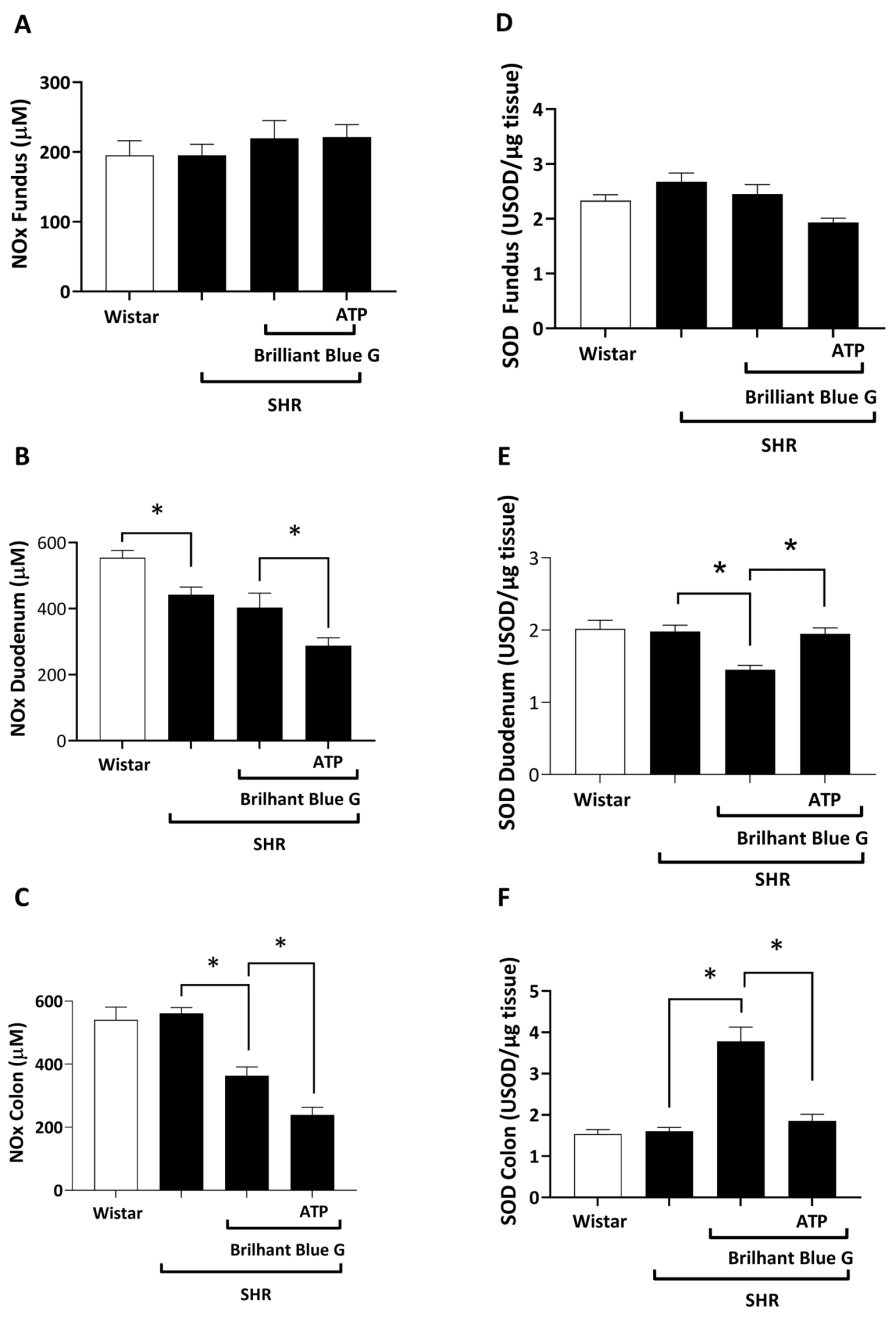
Nitrite/nitrate (NOx) concentrations in the gastric fundus (**A**), duodenum (**B**), and colon (**C**) of Wistar rats *vs* spontaneously hypertensive rats (SHR), SHR treated with BBG, and SHR treated with BBG plus ATP. Superoxide dismutase (SOD) activity in the gastric fundus (**D**), duodenum (**E**), and colon (**F**) of Wistar rats *vs* SHR, SHR treated with BBG, and SHR treated with BBG plus ATP. Data are reported as means±SE. *P<0.05 (one-way ANOVA, followed by the Tukey test). BBG: Brilliant Blue G; ATP: adenosine triphosphate; USOD: units of SOD.

**Figure 6:**
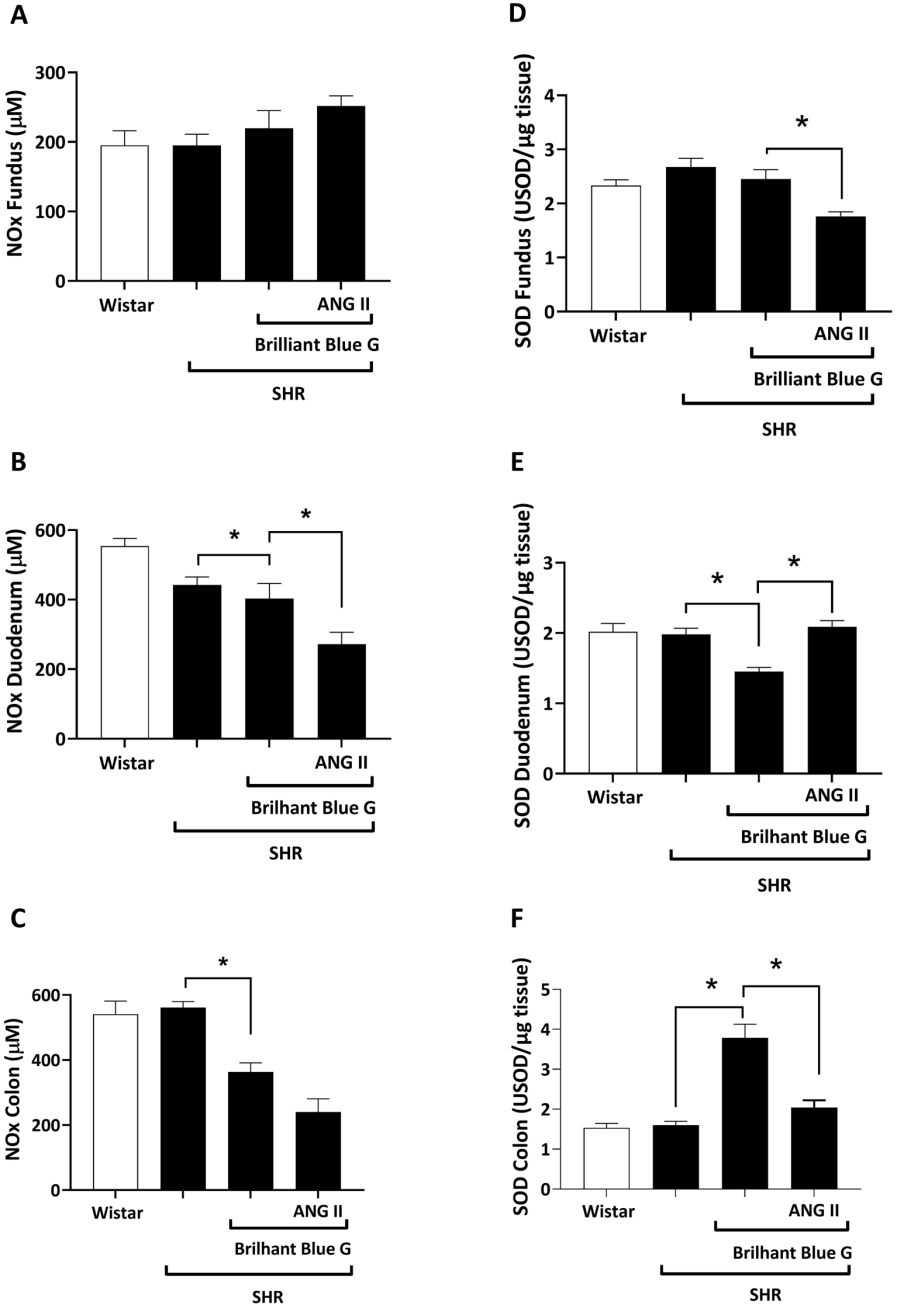
Nitrite/nitrate (NOx) concentrations in the gastric fundus (**A**), duodenum (**B**), and colon (**C**) of Wistar rats *vs* spontaneously hypertensive rats (SHR), SHR treated with BBG, and SHR treated with BBG plus ANG II. Superoxide dismutase (SOD) activity in the gastric fundus (**D**), duodenum (**E**), and colon (**F**) of Wistar rats *vs* SHR, SHR treated with BBG, SHR treated with BBG plus ANG II. Data are reported as means±SE. *P<0.05 (one-way ANOVA, followed by the Tukey test). BBG: Brilliant Blue G; ANG II: angiotensin II USOD: units of SOD.

The concentration of NOx did not significantly change in the gastric fundus (195.20±16.07 µM vs 195.20±20.81 µM; Figure 5A) and colon (561.50±18.11 µM *vs* 541.00±40.48 µM; Figure 5C) of SHR compared to control rats, but it slightly decreased (P<0.05) in the duodenum (442.40±22.78 *vs* 554.10±22.17 µM; Figure 5B) of the same animals. The P2X7 receptor antagonist, BBG, decreased (P<0.05) the concentration of NOx in the colon of SHR (364.00±27.12 *vs* 561.50±18.11 µM; Figure 5C), but it had no effect in the gastric fundus (Figure 5A) and duodenum (Figure 5B) of the same animals. Administration of ATP on top of BBG further decreased (P<0.05) the concentration of NOx in the duodenum (287.60±24.19 *vs* 403.40±43.09 µM; Figure 5B) and colon (239.30±23.81 *vs* 364.00±27.12 µM; Figure 5C), but not in the gastric fundus (Figure 5A) of SHR animals. Likewise, we observed a similar activity pattern when ANG II was used instead of ATP (Figure 6A, B, and C). The concentration of NOx did not significantly change (P>0.05) in the gastric fundus of SHR animals after treatment with BBG (219.70±25.49 µM) or BBG+ANG II (252.00±14.51 µM). Yet, ANG II further decreased NOx levels in the duodenum (272.20±34.03 *vs* 403.40±43.09 µM; Figure 6B) and in the colon (240.10±40.82 µM vs 364.00±27.12 µM; Figure 6C) of SHR treated with BBG.

The activity of SOD (USOD/µg tissue) was not different in the gastric fundus (Figure 5D), duodenum (Figure 5E), and colon (Figure 5F) of SHR and control groups. Treatment with BBG did not change the SOD activity in the gastric fundus of SHR animals, but it either decreased or increased the activity of this enzyme in the duodenum (1.45±0.06 *vs* 1.98±0.08 USOD/µg tissue; Figure 5E) and colon (3.78±0.34 *vs* 1.60±0.09 USOD/µg tissue; Figure 5F), respectively, of these animals. Administration of ATP to SHR animals treated with the P2X7 receptor antagonist, BBG, restored the SOD activity in the duodenum (1.949±0.081 *vs* 1.981±0.088 USOD/µg tissue; Figure 5E) and colon (1.85±0.158 *vs* 1.60±0.09 USOD/µg tissue; Figure 5F) to the levels obtained in untreated animals. Administration of ANG II together with BBG to SHR animals also restored the activity of SOD in the duodenum (2.08±0.08 *vs* 1.98±0.08 USOD/µg tissue; Figure 6E) and colon (2.04±0.17 *vs* 1.60±0.09 USOD/µg tissue; Figure 6F) of these animals. ANG II decreased (P<0.05) the activity of SOD in the gastric fundus of SHR animals treated with BBG (1.76±0.08 *vs* 2.45±0.17 USOD/µg tissue; Figure 6D) and a similar tendency (P>0.05) was also observed when ATP was used instead of ANG II (Figure 5D). Moreover, the linear regression between GE and oxidative stress is shown in Supplementary Figures S1, S2, S3, and S4.

## Discussion

Data from this study showed that spontaneously hypertensive rats (SHR) have delayed solid GE and present significant changes in oxidative/nitrosative stress and inflammation biomarkers, namely in the MPO activity, in the gastric fundus compared to control littermates. Subcutaneous administration of the P2X7 receptor antagonist, BBG, fully reversed these changes. Co-administration of ATP or ANG II abrogated the protective effect of BBG. These findings uncovered a yet unexplored putative interplay between the purinergic signaling cascade and the renin-angiotensin system underlying GI tract disturbances in hypertensive individuals, which might be therapeutically relevant.

Administration of liquid and/or solid meals is instrumental to assess the GE rate. Liquids are useful to characterize the initial accelerating phase of the GE (17). Solid GE begins with the comminution of an ingested meal in the antral portion of the stomach, after which the solid particles pass to the liquid phase, suggesting that solid GE depends on the gastroduodenal pressure gradient (18). Solid GE is regulated mainly by the tonic resistance of the pyloric sphincter and by the coordinated contractions from the antrum towards the pylorus, which seems to be critical for propelling the gastric content to the duodenum during the emptying phase (18).

Gastrointestinal (GI) dysmotility caused by 5-FU intestinal inflammation has significant repercussions in GE. Accordingly, intestinal inflammation is associated with abnormalities in the control of GI motility, not only at the site of inflammation but also at distant non-inflamed locations (19). In this sense, the levels of oxidative/nitrosative stress biomarkers increase in various GI tract disorders, like acute pancreatitis, reflux esophagitis, inflammatory bowel disease, and gastric dysmotility induced by cancer chemotherapy. The production of free radicals causes inhibition of the GE in rats, and this effect is reversed by free radical scavengers (e.g. vitamin C, vitamin E, glutathione, tiopronin) and by 5-HT_3_ antagonists (e.g. ondansetron). Free radical generation leads to the release of serotonin (5-HT) by mucosal cells of the GI tract, which in turn stimulates the peripheral 5-HT_3_ receptors on vagal afferents causing relaxation of stomach smooth muscle resulting in delayed GE (20).

Here, we assessed the GE rate of a solid meal in hypertensive animals while previous accounts focused mostly on semi-solid or liquid GE (21). Our data showing that GE is delayed in SHR is in agreement with that of Hatanaka et al. (21) using a semi-solid test meal in the same animal model. Likewise, Lima et al. (15) obtained similar results showing that the liquid GE rate is also delayed in the 2K1C hypertension model. An imbalance of the autonomic nervous system activity is the most probable explanation for the GE delay in hypertensive animals. The sympathetic activity overshooting that is commonly observed in hypertensive individuals may be responsible for inhibition of the GI motility resulting in decreases in GE. Alternative explanations may involve decreases in the parasympathetic/vagal tone leading to impaired gastric motility and secretion (22).

Deregulation of the RAAS is commonly implicated in hypertension (23). It is also worth noting that sympathetic overstimulation promotes the release of renin from the juxtaglomerular apparatus, thus contributing to strengthening and enduring RAAS activation. Upon increasing tissue concentrations of ANG II, activation of AT1 and AT2 receptors in the gastric mucosa may contribute to changes in stomach motor activity (24). Activation of AT1 receptors induces gastric vasoconstriction, thus resulting in deficits in NO production and activity with a direct impact on the GE rate (25).

This study was designed to investigate the role of the P2X7 purinoceptor activation on solid GE and oxidative/nitrosative stress-related biomarkers in SHR animals using BBG, a nonselective P2X7 receptor antagonist (26). Our findings showed that blockage of the P2X7 receptor rehabilitated the GE of a solid meal and normalized oxidative/nitrosative stress and inflammatory biomarkers in SHR animals. This indicates that the P2X7-mediated purinergic signaling cascade is an important player in GE deficits associated with hypertension. This theory is strengthened because exogenously applied (IP) ATP revoked the beneficial effects of BBG on solid GE in SHR. Unexpectedly, we also observed a reversal of the BBG beneficial effect on SHR animals when ANG II was used instead of ATP. While ATP, the endogenous ligand of the P2X7 receptor, is implicated in inflammation, oxidative stress, and GI tract motility, ANG II may act via AT_1_ receptors to further modify GI motility, produce sympathetic overactivity, and promote inflammation and oxidative stress (27). Thus, our results suggested here for the first time that the purinergic signaling cascade and the RAAS interplay caused GI motility deficits in hypertensive animals, which may open new avenues for novel therapeutic interventions.

Oxidative stress is an important trigger of ATP release by endothelial cells, which in turn may act as an autocrine/paracrine messenger, via P2 purinoceptors activation, to promote intracellular Ca^2+^ waves in neighboring cells (4). ATP acting via ionotropic P2X7 receptors promotes inflammation and increases the release of inflammatory cytokines (28). In the GI tract, inflammation favors glial cell proliferation and the production of cytokines, which actions may be potentiated and endured by P2X7 receptors activation of the inflammasome (29).

Hypertension has been associated with increased tissue inflammation and ROS generation, thus contributing to oxidative stress in target organs. However, the role of hypertension on inflammation and oxidative stress affecting the GI tract still lacks consistent experimental evidence. Our research group was pioneering to show increases in pro-inflammatory cytokines and pro-oxidant markers in the duodenum of rats with 2K1C-induced hypertension (12). Here, we aimed at further characterizing the alterations of oxidative/nitrosative stress and inflammatory biomarkers in the gastric fundus, duodenum, and colon of SHR rats, which might have a functional impact on GI motility, namely on solid GE.

The MPO activity reflects the number of neutrophils infiltrating tissues and, therefore, may be used as an oxidative stress marker (30). In the GI tract, MPO activity increases in parallel to IL-8 production by tissue macrophages, which facilitates the recruitment of neutrophils (31). Here, we show that the MPO activity increased in the gastric fundus of SHR compared to control rats. This is compatible with the theory that hypertension causes inflammation and oxidative stress leading to infiltration of the stomach by MPO-containing neutrophils (26,27). Blockage of P2X7 receptors with BBG fully prevented excessive MPO activity in the gastric fundus of SHR animals, indirectly indicating that this compound tempered hypertension-induced gastric inflammation and leukocyte recruitment (32). No significant changes were observed in the MPO activity in the duodenum and colon among SHR and control rats; administration of BBG with or without ATP or ANG II was also devoid of effect in both intestinal segments. These results raise the hypothesis that BBG may act in these GI sub-regions via different purinoceptor subtypes, ATP metabolites, and/or opening of calcium channels (33); all these hypotheses deserve experimental confirmation in future studies.

The levels of nitrite/nitrate (nitrogenous reactive metabolites, NOx) did not significantly differ in the gastric fundus and colon of SHR compared to control rats, but we noticed a slight reduction of NOx in the duodenum of SHR animals. The tissue levels of NOx are proportional to the activity of nitric oxide synthase (NOS) and the formation of peroxynitrite resulting from the activation of tissue macrophages (34). Dietary nitrate is partially metabolized in the stomach and may also contribute to NOx levels in the tissue. Therefore, one may conclude that the delay in solid GE observed in SHR animals can hardly be attributed to changes in NO production in our experimental settings (35).

The endogenous antioxidant defense in the GI tract includes GSH and SOD, which provide strong antioxidant effects against ROS and protect tissues from cell damage induced by oxidative intermediates (36). In this context, SOD merits special attention since its final enzymatic function results in the reduction of reactive oxygen and nitrogen species (37), suggesting that it may be a marker of oxidative stress. No changes in SOD were observed in the gastric fundus, duodenum, and colon of SHR compared to their control littermates. Nevertheless, the SOD activity differed significantly in these tissues upon administration of BBG; while the P2X7 receptor antagonist decreased the SOD activity in the duodenum, it promoted this enzyme activity in the colon. Interestingly, both ATP and ANG II reversed the changes in SOD activity caused by BBG in the duodenum and colon of SHR animals. These results agreed with previous findings suggesting that ANG II (and probably ATP) may affect the antioxidant balance of distinct segments of the GI tract differently via the activation of AT1 receptors (38).

To evaluate the effects of hypertension on the sulfhydryl antioxidant activity of the GI tract of SHR animals, we measured the tissue pool of reduced glutathione, GSH. Data showed that GSH levels were significantly reduced in the duodenum and colon of SHR compared to their control littermates, but no changes were observed in the gastric fundus of the same rats. Subcutaneous administration of BBG counteracted hypertension-induced GSH deficits in the duodenum and colon of SHR animals. The preventive effect of the P2X7 receptor antagonist disappeared when it was used together with ATP or ANG II. Surprisingly, BBG caused a marked reduction in GSH in the gastric fundus of SHR. This paradoxical effect may be interpreted considering that BBG may exert pro-oxidant and anti-oxidant effects since the antioxidant protection of reduced GSH in the GI tract is intimately related to NADPH consumption and hydroxyl production (39). Moreover, GSH also confers protection against gastric acid secretion (35). Considering that, gastroparesis in SHR can increase gastric acid secretion and that BBG can reverse gastroparesis (see above), the P2X7 receptor antagonist can indirectly reduce the gastric acid secretion and, thereby, the requirement for the conversion of oxidized glutathione to GSH. This hypothesis is supported by the increase of the GSH pool in the gastric fundus of SHR animals treated with BBG plus ATP or Ang II.

Data from this study prompted the hypothesis that inflammation and oxidative/nitrosative stress pathways may be involved in GE delay in SHR rats. GSH and NO are major regulators of GE according to Pérez et al. (35). ROS/RNS may also account for the regulation of GE via a complex mechanism that involves i) the production and release of gasotransmitters, such as NO (produced by NOS), and ii) the participation of antioxidant factors, such as GSH and related enzymes, which control the generation of reactive species and provide mucosal protection against gastric acid secretion (13). In this regard, our findings showed that the amount of NOx positively correlated with the GE rate, both in control and SHR animals. Yet, this correlation disappeared or was reversed when SHR were treated with the P2X7 receptor blocker, BBG, either alone or in combination with ATP or ANG II, respectively. Though not significant, we observed a tendency for a negative correlation between solid GE rate and the MPO activity, which was steeper in the gastric fundus of control and SHR+BBG animal groups compared to untreated SHR animals. Previous reports about diabetic gastroparesis suggest that ROS and inflammation can cause gastric dysmotility (40). Here, we showed that hypertension-induced delayed GE might also be related to low-grade inflammation and oxidative/nitrosative stress. These findings also suggested the participation of ANG II and P2X7 receptor in the modulation of these effects.

**Figure 7:**
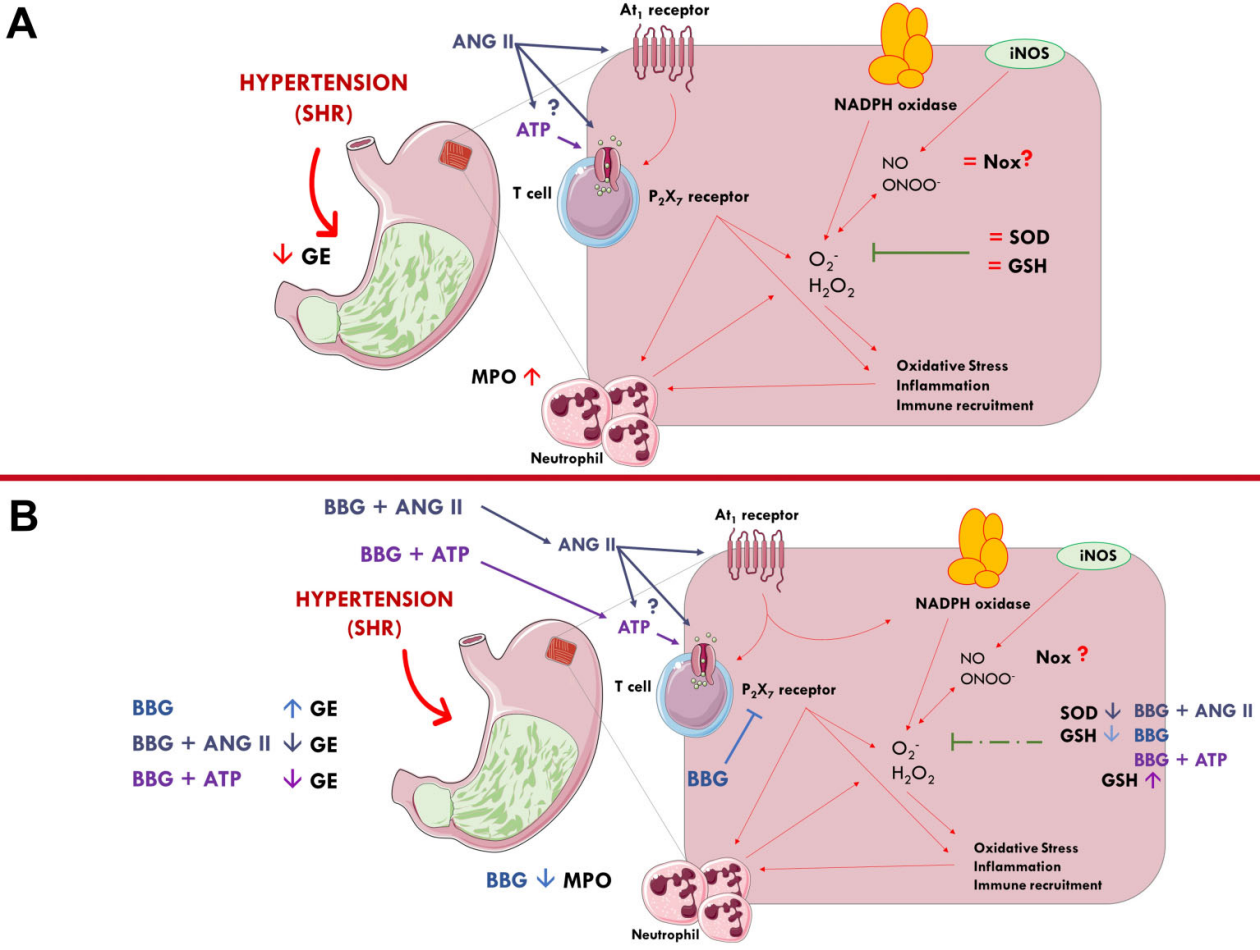
Proposed mechanism of hypertension effects on gastric emptying (GE) and oxidative stress in the gastric fundus. In spontaneously hypertensive rats (SHR), there was a delay in gastric emptying rate, probably due to oxidative stress and inflammation caused by hypertension. Hypertension-induced increases in ANG II acting via the G protein-coupled AT1 receptor subtype favors NADPH oxidase and iNOS activity, which triggers oxidative/nitrosative stress and the release of reactive oxygen and nitrogen species, along with inflammatory lymphocyte recruitment and differentiation. Here, we hypothesized that ANG II may act synergistically with P2X7 receptors located in lymphocytes and other cells by promoting the local release of the danger molecule ATP. Synergism between ANG II and the purinergic system may be either via a direct mechanism or indirectly by increasing inflammatory stress. This inflammatory cascade triggered by ANG II and purinoceptors activation acting together with sympathetic overactivation in hypertensive animals may contribute to the recruitment of immune cells leading to a further increase in neutrophil infiltration and MPO overshooting. No alterations were observed in the activity of SOD and the levels of GSH and NOx in SHR. Blockage of P2X7 receptors with BBG increased the GE rate and reduced both the MPO activity and GSH levels. Interestingly the effect of BBG was “de novo” abrogated by both ATP and ANG II suggesting a crosstalk between ATP-sensitive P2X7 receptors and AT1 receptors activation in this endeavor. ANG II: angiotensin II; AT1: angiotensin II receptor type 1; ATP: adenosine triphosphate; BBG: Brilliant Blue G; GSH: glutathione; iNOS: inducible nitric oxide synthase; NADPH: adenine and nicotinamide dinucleotide phosphate; NE: norepinephrine; MPO: myeloperoxidase; SHR: spontaneously hypertensive rats; SOD: Superoxide dismutase. Red arrows indicate hypertension effects. Green lines indicate antioxidant protection. Light blue arrows indicate BBG actions. Dark blue arrows indicate BBG + ANG II actions. Purple arrows indicate BBG + ATP actions.

In conclusion, the data showed for the first time a novel interaction between the purinergic signaling cascade and the renin-angiotensin system underlying solid GE delay was detected in hypertensive animals (Figure 7). The participation of oxidative/nitrosative stress and inflammatory mediators was also hypothesized, yet the putative clinical repercussions of our findings require further studies to elucidate more deeply the cellular and molecular components of these phenomena.

## Supplementary Material

Click here to view [pdf].
